# Saving neonatal lives by improving infection prevention in low-resource units: tools are needed

**DOI:** 10.7189/jogh.09.010319

**Published:** 2019-06

**Authors:** Julia Johnson, Ibukunoluwa C Akinboyo, Melanie S Curless, Aaron M Milstone, Susan E Coffin

**Affiliations:** 1 Department of Pediatrics, Division of Neonatology, Johns Hopkins University School of Medicine, Baltimore, Maryland, USA; 2 Department of Pediatrics, Division of Pediatric Infectious Diseases, Duke University, Durham, North Carolina, USA; 3 Department of Hospital Epidemiology and Infection Control, The Johns Hopkins Hospital, Baltimore, Maryland, USA; 4 Department of Pediatrics, Division of Pediatric Infectious Diseases, Johns Hopkins University School of Medicine, Baltimore, Maryland, USA; 5 Department of Pediatrics, Division of Infectious Diseases, University of Pennsylvania, Philadelphia, Pennsylvania, USA

Globally, neonatal mortality rates remain relatively stagnant despite overall progress in reducing under-5 mortality [1]. In regions with highest mortality for neonates, infections account for up to 30%-50% of deaths [2]. In many low and middle income countries (LMICs), births within health care facilities are encouraged as a mechanism to reduce both maternal and neonatal mortality [1]. However, the resulting increased demand for facility births has not been accompanied by comparable increases in capacity for delivering quality care and enhancing the safety of maternal and neonatal patients. Shortages in space, trained staff, and consumable resources have frayed many maternal-neonatal health care settings. To date, there have been limited systematic efforts to improve quality of care, while demands on facilities expand.

Significant gaps exist in infection prevention and control (IPC) practices in maternal-neonatal care settings, resulting in increased risk of health care-associated infections (HAI) for both mother and baby [3]. Healthcare facilities in LMICs are providing increasingly complex care to high-risk mothers and neonates with growing numbers of Special Care Nurseries and Neonatal Intensive Care Units (NICUs). Facility-based care in LMIC settings ranges from nurse-led units in remote areas with minimal access to invasive devices, medications, and imaging or microbiological support services to those with capacity for more advanced and invasive technology. Common features include challenges with existing space and infrastructure, lower than recommended staff-to-patient ratios, inability to identify HAI with accompanying feedback of data, difficulty implementing improvement strategies, frequent shortages of equipment and supplies, and lack of IPC support, guidelines, and education. Deficiencies among the World Health Organization (WHO) Core Components of Infection Prevention and Control Programs lead to gaps in basic IPC practices in maternal and neonatal settings such as poor hand hygiene, lack of aseptic technique, improper reprocessing of multi-use equipment, and inadequate environmental cleaning pose significant risks to hospitalized neonates, who are especially vulnerable to HAI due to factors such as immature immune systems, poor skin integrity, and need for life-sustaining invasive procedures [4]. Practices unique to neonatal care include umbilical catheter placement, surfactant administration, isolette and radiant warmer use, preparation and storage of maternal and donor breast milk as well as infant formula, and kangaroo care; each of these practices requires special consideration to ensure appropriate IPC practices.

**Figure:**
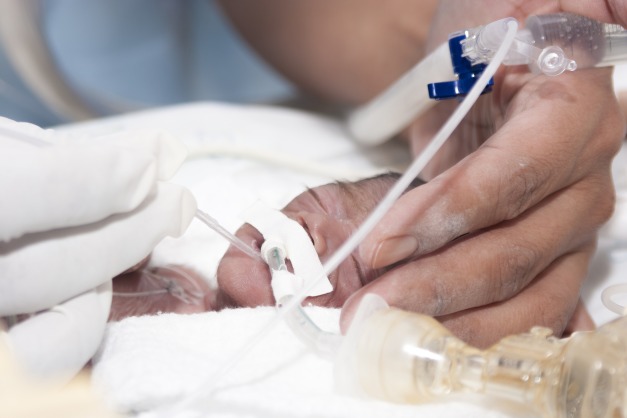
Photo: istock.com/herjua.

In many resource-limited settings, neonatal sepsis is predominantly caused by Gram-negative organisms, with high prevalence of antimicrobial resistance [4,5]. A recent study conducted in India highlighted high rates of both early- and late-onset sepsis in hospitalized neonates, caused predominately by Gram-negative organisms such as *Acinetobacter* species (spp) and *Klebsiella* spp [5]. To reduce neonatal HAI and associated mortality, research is needed to elucidate reservoirs and mechanisms of transmission of these organisms outside of outbreak settings and ways in which facilities can optimize implementation of IPC in obstetric and neonatal units [6].

As contributors to HAI risk are variable and IPC resources are limited, standardized assessments linked to improvement strategies are essential to guide health care facilities in LMIC to prioritize high-impact strategies to reduce neonatal HAI and death. The WHO has designed several tools to assess hospital-wide IPC practices, including the Hand Hygiene Self-Assessment Framework and the Infection Prevention and Control Assessment Framework at the Facility Level (IPCAF) [7,8]. Strengths of IPCAF include its design as a self-assessment tool, the inclusion of core IPC components, and its linkage to the Interim Practice Manual, a resource that can be used to strengthen IPC activities [8]. This self-assessment tool assigns an “IPC level”, ranging from “inadequate” to “advanced”. However, IPCAF does not provide comprehensive assessments of IPC practices unique to maternal and neonatal care (**Table 1**). Similarly, the Water and Sanitation for Health Facility Improvement Tool (WASH FIT), a WHO continuous improvement framework, does not have specific maternal-neonatal content [10].

**Table 1:** Summary of critical gaps of current IPC assessment tools

Assessment tool	Gaps	Examples
ICAT, 2nd edition (2009) [9]	NICU-specific content	- ICU module focused on adult care - Labor & Delivery module incorporates only basic neonatal care
	Evidence-based recommendations	- Modules assessing needle and sterile glove reprocessing practices - Hexachlorophene listed as acceptable topical antiseptic agent in neonates
	Antimicrobial stewardship principles	- Scoring system rewards use of prophylactic antibiotics for C-sections but does not account for improper prolonged antibiotic use
	Validation of self-reported performance	- Checklists for direct observation limited to hand hygiene, injection administration, and waste management following deliveries
	Layout and format	- Lengthy paper format
	Linkage to improvement content	- Each module followed by annotations summarizing best practices - Scores and grades assigned without linkage to educational content or tools for creation of action plan
WASH FIT (2018) [10]	Maternal- and neonatal-specific focus	- Tool designed for primary and secondary health care facilities without IPC content specific to maternal and neonatal units or care of premature and critically ill neonates
WHO Hand Hygiene Self-Assessment Framework (2010) [7]	IPC focus beyond hand hygiene assessment	- Comprehensive assessment using 5 components and 27 indicators, but limited to hand hygiene practices
WHO IPCAF at the Facility Level (2018) [8]	Maternal- and neonatal-specific focus	- 8 core components at facility level, no specific assessments focused on inpatient care of maternal and neonatal patients

C-section – Cesarean section, ICAT – Infection Control Assessment Tool, IPC – infection prevention and control, IPCAF – Infection Prevention and Control Assessment Framework, NICU – neonatal intensive care unit, WASH FIT – Water and Sanitation for Health Facility Improvement Tool, WHO – World Health Organization

The United States Agency for International Development (USAID) sponsored Infection Control Assessment Tool (ICAT) is a more comprehensive tool designed to assess IPC practices across acute care hospitals. It includes 22 modules as well as checklists for direct observation of several key practices [9]. Strengths of the ICAT include its modular composition and comprehensive facility-wide scope. However, the tool is insufficient for assessing neonatal and maternal IPC practices. To understand common IPC gaps, we conducted serial assessments of maternal and neonatal practices using the ICAT at five LMIC facilities in Malaysia and India and noted the following: (1) lack of NICU-specific content; (2) outdated recommendations; (3) insufficient validation of self-reported performance; (4) format not conducive to efficient data collection; and (5) lack of linkage to improvement content (**Table 1**).

These findings suggest that an assessment tool targeting facility-based maternal and neonatal IPC practices is needed and should address several key factors. First, an assessment tool must cover the wide spectrum of care provided for mothers and neonates in LMICs. Fundamental elements of IPC practice, such as hand hygiene, must be incorporated, but advanced care content, such as IPC related to the use of invasive devices and prolonged supportive care in neonates, should also be included for use by sites where these interventions are provided. Components of the tool focusing on antibiotic administration should also embrace antimicrobial stewardship principals targeting areas with high endemic rates of antimicrobial resistance. While self-assessment would facilitate broad uptake in LMICs, direct observation of key IPC practices by a trained assessor, whether internal or external, would ensure observations made had a high probability of guiding true gaps in IPC practice. An assessment tool should highlight strengths and opportunities for improvement, guiding facilities to plan and implement interventions with the ultimate aim of creating a sustained improvement in IPC practices. Linkage to educational materials and implementation tools would strengthen such a tool, providing key recommendations to health care facilities seeking to improve IPC in maternal and neonatal care. Ultimately, a comprehensive assessment tool designed specifically for maternal and neonatal care will allow facilities to most effectively reduce morbidity and mortality due to HAI in this vulnerable population.
